# On the Rheological Properties and Printability of Sodium Alginate–Carboxymethyl Chitosan Composite Solutions for Tissue Scaffold Printing

**DOI:** 10.1002/bip.70050

**Published:** 2025-09-24

**Authors:** Xavier L. Tabil, Tate N. Cao, Xiongbiao Chen

**Affiliations:** ^1^ Division of Biomedical Engineering College of Engineering, University of Saskatchewan Saskatoon Saskatchewan Canada; ^2^ Ron & Jane Graham School of Professional Development College of Engineering, University of Saskatchewan Saskatoon Saskatchewan Canada; ^3^ Department of Mechanical Engineering College of Engineering, University of Saskatchewan Saskatoon Saskatchewan Canada

**Keywords:** composite biomaterials, extrusion printing, flow rate modeling, printability, rheological properties, tissue scaffolds

## Abstract

Composites of sodium alginate (Alg) and carboxymethyl chitosan (CMCS) are used to 3D print tissue scaffolds, but the rheological properties and printability of these composites remain underreported, resulting in time‐consuming trial‐and‐error printing. This study investigates these properties to rigorously design the 3D printing process. Dynamic shear tests characterize viscoelastic and frequency‐dependent properties, while steady shear tests assess the apparent viscosity and temperature‐dependent viscosity. A novel approach based on mass flow rate models guides the printing of two‐layer scaffolds for printability analysis. Brightfield microscopy and printability indexes quantify the deviations between printed and designed scaffolds, defined as printability. Results show that Alg predominantly directs the rheological properties. At 4% w/v Alg, the addition of < 3% w/v CMCS reduces elasticity, contrary to the trend where increasing CMCS increases elasticity. CMCS improves the thermal resistance of the composites, while Alg reduces it. Of the composites printed, a 4% w/v Alg + 1% w/v CMCS formulation most accurately replicates the designed scaffold, and adding CMCS improves scaffold printing repeatability by at least threefold compared to Alg‐only solutions. These findings provide a framework that informs the preparation and performance of Alg‐CMCS composites with tunable properties, advancing scaffold bioprinting for tissue engineering.

## Graphical abstract

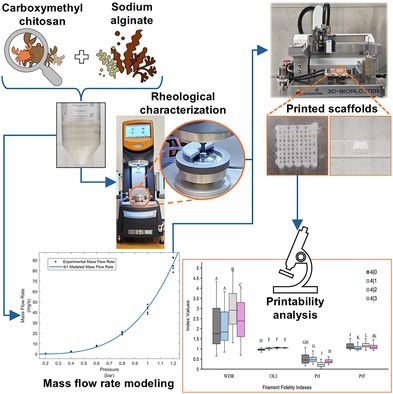

## 1 Introduction

Tissue scaffolds are engineered support structures currently fabricated by extrusion three‐dimensional (3D) printing polymers. Scaffolds provide architectural, mechanical, and biological support that aids cells in regenerating damaged or defective tissue [1, 2]. Polymers are commonly used to print scaffolds because of their biodegradability, biocompatibility, and ability to form hydrogels [3, 4, 5]. Hydrogels are gelled polymers that contain a large amount of water compared to their total mass, which is useful in replicating the cell‐friendly environment present within living beings. Sodium alginate (Alg) and carboxymethyl chitosan (CMCS) are natural sources of polymers that can form hydrogels and are commonly used in food and cosmetic industries, clearly demonstrating their potential for use in biomedical applications. Alg is derived from brown seaweed and is commonly used in biomedical applications because of its hydrophilicity, shear‐thinning behavior, biocompatibility, and ease of crosslinking to form hydrogels by ionic exchange in the presence of cations like calcium [6, 7, 8, 9]. Like Alg, CMCS is most commonly obtained from marine sources, specifically crustacean shells, and has high potential for biomedical applications because of its mucoadhesive, antimicrobial, biocompatible, and cell adhesion properties [8, 9, 10, 11].

Extrusion 3D printing an Alg‐CMCS solution is performed by applying pneumatic pressure through a syringe directly into a crosslinking medium, for example, calcium chloride, which allows the Alg‐CMCS solution to rapidly harden in the presence of calcium ions [2, 3, 4, 5, 6, 7, 8, 9, 10, 11, 12, 13, 14]. This is the mechanism by which larger 3D printed structures are formed. The printing of tissue scaffolds requires an understanding of the rheological properties and printability of the polymeric printing materials. The rheological properties of biomaterial solutions are important because they encompass the flow behavior of the material during the printing process. The ability to flow and the behavior of the flow under pressure are affected by the printing materials and can influence the printing resolution [15, 16]. Printing resolution falls under the umbrella of printability, which is defined as the ability for materials to accurately 3D print a structure from a design and its capability of maintaining the structure after printing [17, 18]. Printability can be characterized in terms of indexes of extrudability, filament fidelity, and structural integrity. Poor printability could lead to mechanical weakness or even complete structural failure, destroying the scaffold. In addition to the rheology and composition of biomaterial solutions, the printability of a scaffold is affected by factors such as the complexity of the scaffold design, the dimensions of the printing needle, and the extrusion printing parameters [17, 19]. A material suitable for extrusion printing leverages the shear‐thinning viscoelastic behavior observed in printable polymers, while balancing the rheology of the material and the printing parameters required to create the scaffold.

The rheological properties and printability of Alg and CMCS‐containing solutions for the formation of scaffolds are of equal importance to properties like the mechanical and biological properties of scaffolds. The combination of rheology and printability affects the architectural properties of the tissue scaffolds [20, 21]. Without sufficient architectural support, tissue scaffolds would have no mechanical or biological benefits to contribute to cells in the regeneration of tissue [22, 23]. Occasionally, studies leave the rheology and/or printability of scaffold materials briefly described or leave them unexamined while focusing only on the biological properties or mechanical properties [24, 25, 26, 27, 28]. Because the composition of biomaterials affects the rheology and consequently the printability, these two properties should also be well defined in research pertaining to tissue scaffolds. An Alg‐CMCS composite should benefit from the positive properties of both materials, but the components may interact with each other in unexpected ways, and the composite would have new characteristics, unpredictable by looking at an Alg‐only or CMCS‐only solution. While composite solutions containing Alg and CMCS have been printed previously, to our knowledge, the interactions and effects of both components on the rheological properties and printability of Alg‐CMCS solutions have not been thoroughly researched.

Aside from the time‐consuming trial‐and‐error approach, printing pressure and printing speed can be mathematically determined in two ways. One method is to make use of the flow behavior, obtained from a rheometer, to calculate printing parameters. While data have been used to relate rheometer‐derived viscosity to printing flow behavior [29], it should be noted that the flow behavior of composite solutions is highly sensitive to temperature. Since rheometers and bioprinters often operate under different temperature control systems, there may be a difference in the precision of applied temperature between the rheometer for steady shear tests and the printer for fabricating the scaffolds. Even slight differences in thermal conditions can introduce significant model errors, and the printing parameters determined based on the flow behavior obtained from the rheometer would not be applicable for use in printing. Therefore, this study uses an alternative approach: using simple extrusion experiments to obtain fluid properties that can be modeled and used to calculate printing parameters [30, 31]. This method offers a more direct and practical link between material behavior and printing performance. To the best of our knowledge, this is the first application of a tapered needle, mass flow rate model to determine printing parameters for the fabrication of tissue scaffolds. This novel modeling method enables rapid, rheometer‐free characterization of the printable solutions, improving reproducibility and accessibility in scaffold printing.

In this study, we present Alg‐CMCS composite solutions and the methods used to investigate the impact of Alg and CMCS variation on the rheological properties and the printability of the solutions for tissue scaffold fabrication. The influence of Alg and CMCS on the rheological properties of the composites was discovered by employing dynamic and steady shear tests and characterized in terms of linear viscoelastic region (LVR), storage modulus (*G*′), and loss modulus (*G*″), temperature effect on the viscosity at constant shear rate, and so forth. Building on the modeling approach mentioned above, we developed a novel approach to define the printing pressure and horizontal moving speed of the needle (referred to as printing speed) for 3D scaffold fabrication. This mass flow rate model removed the need for the trial‐and‐error approach typically reported in the literature. Using this method, we analyzed the effect of CMCS concentration on scaffold printability and repeatability, quantified using filament fidelity indexes.

## 2 Materials and Methods

### 2.1 Composite Solution Preparation

For rheological testing, sodium alginate (cat# 177775000, Thermo Scientific Chemicals, USA) and carboxymethyl chitosan (cat# sc358091, Santa Cruz Biotechnology, USA) dry powders were manually stirred until homogenous, combined with distilled water, and mixed in a rotator at room temperature overnight. Twelve solutions were prepared by combining 2%, 3%, 4% w/v Alg with 1%, 2%, 3%, 4% w/v CMCS. The synthesized solutions were designated with the following notation, for example, 2|1, meaning 2% w/v Alg combined with 1% w/v CMCS. For printability characterization, four solutions were prepared using the above procedure with 4% w/v Alg and 0%, 1%, 2%, 3% w/v CMCS.

### 2.2 Rheological Tests

The 12 solutions were tested in a Discovery‐series Hybrid Rheometer 20 (HR20) from TA Instruments (USA). The HR20 was set up using both parallel plate (60 mm diameter, gap height 600 μm) and cone‐and‐plate (40 mm, 2° angle, truncated gap 51 μm) configurations for dynamic and steady shear tests, respectively. A bottom, advanced Peltier plate was used for both configurations to precisely control the temperature of the samples. All tests were conducted at a temperature of 25°C, unless otherwise stated. A solvent trap was used throughout testing to prevent drying of the sample, with distilled water as the moisture barrier. Specifics of the dynamic and steady shear tests can be found in Supporting Information.

### 2.3 Printing Parameter Modeling

**FIGURE 1:**
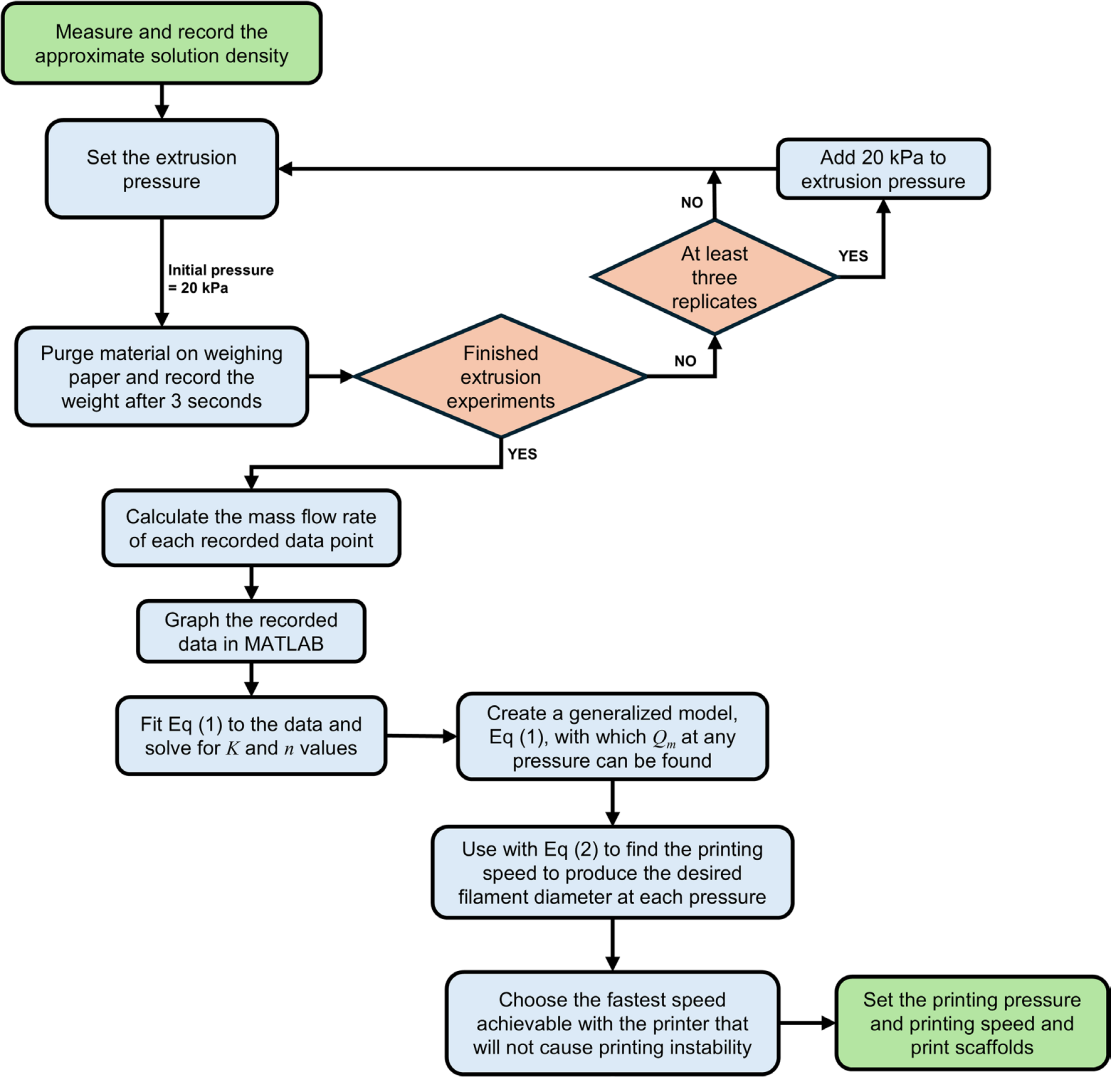
Diagram illustrating how flow rate modeling informs the printing process.

Figure 1 below summarizes the modeling process and outlines how printing parameters, pressure and speed, are determined. Using a generalized flow model for each solution provides flexibility in adjusting the printing parameters, accommodating minor variations. Additionally, creating a mass flow rate model with quick extrusion experiments removes the need for specialized equipment, like rheometers, to link the flow behavior to the design of the printing process.

To model the printing parameters for the EnvisionTEC printer, the mass flow rate was recorded at 20 kPa increments for 3 s between 20 and 120 kPa. This was repeated two more times for each pressure and replicated for each printing solution. The following modified equation was used to model the mass flow rate, *Q*_m_ (mg s^−1^), as a function of pressure: [31, 32]

(1) Qm=ρ·πDi3Do3323n∆P·Di−Do4K·LtDi3n−Do3n1n

where *ρ* is density (mg μL^−1^), which was approximated beforehand; the density of 4|0 was around 1.02 g mL^−1^ while 4|1, 4|2, and 4|3 solutions had an approximate density of 1.1 g mL^−1^. *D*_*i*_ and *D*_*o*_ represent the inlet and outlet diameters (mm), respectively, of the tapered needle. *L*_*t*_ is the length of the tapered section (mm) and *∆P* is the pressure drop within the needle (Pa), which is assumed to equal the input pressure because the syringe diameter is much larger than the diameter of the needle. *K* (Pa·s^*n*^) and *n* are power law variables—consistency index and flow behavior index, respectively—which were determined by curve fitting Equation (1) with the recorded data. By replacing the generalized power law variables with the fitted power law variables, the model solely relied on pressure as an input variable.

With the generalized model, the stress‐free printing speed (*v*_*m*_, mm s^−1^) was calculated for the pressure resolution of the printer (10 kPa) to produce the desired filament diameter, *D*_*f*_ (mm), using: [33]

(2) $$v_{m}=\frac{4Q_{m}}{\pi \rho {D_{f}}^{2}}$$

The fastest printing speeds under 60 mm s^−1^ were chosen along with the corresponding printing pressure to avoid wavering of the printed filaments, which would add printing instability.

### 2.4 Scaffold Design and Fabrication

The printability of Alg‐CMCS solutions was characterized by printing scaffolds with an EnvisionTEC (Germany) 4th Generation 3D‐Bioplotter using a tapered 25‐gauge needle (JG25 1.25HPTTX, Jensen Global Inc., USA). Scaffolds were designed as two‐layer cuboid structures, with a size of 10 mm × 10 mm × 0.4 mm each. The filaments making up the layers were designed to be 200 μm in diameter, with 1 mm spacing between the center of each filament, and a 0° + 90° filament orientation to create square pores, as illustrated in Figure S1. With this filament orientation, horizontal and vertical filaments from a top‐down perspective are referred to as *x* and *y* filaments, respectively.

The previously prepared composite solutions were printed with the modeled printing parameters, as determined above, in a liquid crosslinking solution to gel the printed composite solutions, forming a 3D structure. Briefly, 0.1% w/v polyethyleneimine (PEI, cat# J61270.22, Thermo Scientific Chemicals, USA) was prepared with distilled water. A portion was set aside to be used later, and the rest was combined with calcium chloride dihydrate (cat# 223506, Sigma‐Aldrich, USA) to make 45 mM calcium chloride (CaCl_2_), the crosslinking solution. The reserved 0.1% w/v PEI was used to coat the surfaces of 12‐well plates 24 h before printing to enhance the adhesion of the composite solutions to the surface, increasing fabrication stability. A second crosslinking solution with a concentration of 100 mM CaCl_2_ was prepared with only distilled water to store the scaffolds after printing for at least 10 min before imaging.

### 2.5 Determining Printability With Filament Fidelity Indexes

The printed scaffolds were imaged with brightfield microscopy using a BioTek Lionheart LX automated microscope (Agilent Technologies, USA) and measured using ImageJ v1.53k. The measurements were used to calculate filament fidelity indexes, which quantify the accuracy of the printed scaffold in replicating the designed scaffold. Values closer to 1 indicated the printed scaffold adheres closely to the designed scaffold. Characterizing the ability to print the two fundamental elements of the scaffold, filaments and pores, is important at the primary stage of tissue scaffold fabrication to assess if the physical process is accurate in creating viable scaffolds.

The first filament fidelity index calculated was the width of the printed filament to the designed filament diameter ratio (WDR), where *D*_*P*_ is the width of the printed filament and *D*_*D*_ is the diameter of the filament based on the design: [17]

(3) $$\mathrm{WDR}=\frac{D_{P}}{D_{D}}$$

Similarly, outer length irregularity (OLI) and pore irregularity (PrI) are indexes that compare the length of the filaments and the area of the pores, respectively, between the designed and printed scaffold [17, 18]. OLI quantifies the divergence from the exterior design of a scaffold, while PrI quantifies the divergence of the area of the resulting pores from the design. Rather than using pore size, a one‐dimensional measurement of the empty width or diameter between filaments, PrI was used instead since the pores of the printed scaffolds may be irregularly shaped, so pore size would not accurately depict the porosity of the printed scaffold.

(4) $$\mathrm{OLI}=\frac{{\text{Printed Length}}_{x,y}}{{\text{Designed Length}}_{x,y}}$$

(5) $$\mathrm{PrI}=\frac{{\text{Pore Area}}_{\text{Printed}}}{{\text{Pore Area}}_{\text{Designed}}}$$

Pore fidelity (PrF) was the final index calculated for filament fidelity. PrF describes the shape of the pore itself; in this case, the designed scaffold had perfectly square pores. For this index, a value closer to 1 merely indicates that the shape of the pore resembles a square rather than a rectangle or more irregular shapes.

(6) $$\mathrm{PrF}=\frac{{\left(\text{pore perimeter}\right)}^{2}}{16\times {\text{Pore Area}}_{\text{Printed}}}$$

### 2.6 Data and Statistical Analysis

For viscoelastic characterization, frequency‐dependent behavior characterization, and apparent viscosity characterization experiments, the tests were repeated three times for each solution and on two separate occasions (*n* = 6). Temperature‐dependent viscosity testing was repeated three times on only one occasion (*n* = 3). No statistical analysis was performed on this characterization data, focusing instead on the overall trends and the effect of Alg and CMCS on those trends. For printability optimization, at least four scaffolds were printed from each of the four Alg‐CMCS solutions on two separate occasions (*n* = 8), for a total of 32 scaffolds. Each scaffold was measured at nine different regions. In each region, four measurements were taken for the filament width, 1 for pore area, and 1 for pore perimeter. Additionally, 10 measurements of the outer edge‐to‐edge length were taken for each scaffold. In summary, for each printed scaffold: filament width data (*n =* 36); outer edge‐to‐edge length data (*n* = 10); pore area data (*n* = 9); and pore perimeter data (*n* = 9). These correspond to a total number of *N* = 1152, *N* = 320, *N* = 288, and *N* = 288, statistical samples, respectively.

Statistical analysis on printability was performed using R v4.4.3 through RStudio. A large majority of the data obtained was not normally distributed, so hypothesis testing was performed using Kruskal–Wallis tests on each calculated filament fidelity index. If significance between medians was indicated, Dunn's test was used to conduct pairwise comparisons with a Holm–Bonferroni correction applied to control familywise error.

## 3 Results

### 3.1 Viscoelastic Properties of the Composite Solutions

**FIGURE 2:**
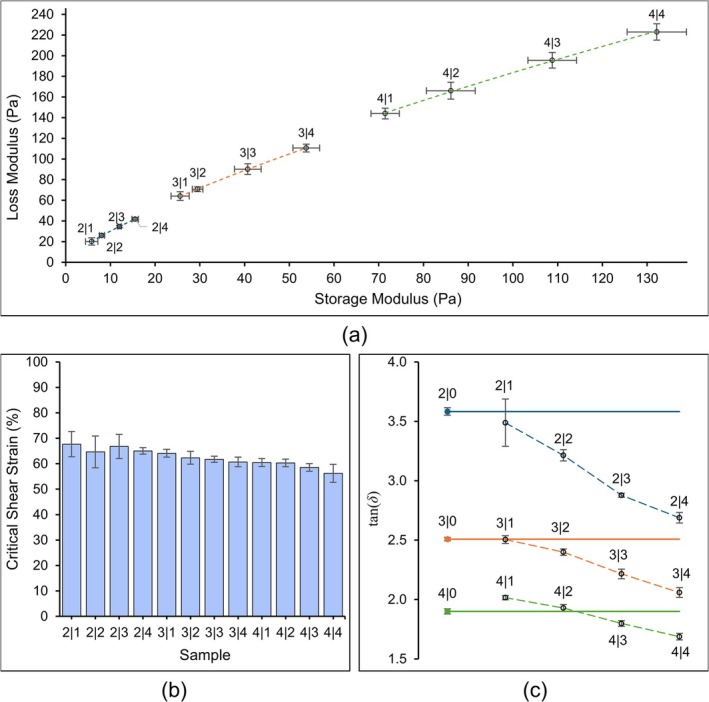
(a) *G*′ and *G*″ values with trendlines, (b) critical strain of all solutions, representing the upper limit of the LVR, and (c) loss tangent for all solutions with horizontal lines for the Alg‐only solutions for comparison. All error bars represent standard deviation from the means, and separate colors show grouping by Alg concentration.

The thresholds of *G*′ and *G*″ increased as the concentration of both Alg and CMCS increased, seen below in Figure 2a. For all solutions, *G*′ was higher than *G*″, and this magnitude decreased as the concentration of both Alg and CMCS increased, from 3.4 times higher and down to 1.7 times higher.

For the Alg‐CMCS solutions, the limit of the LVR was characterized at 61.95% ± 5.73% strain (Figure 2b). Informed by this, subsequent experiments that were strain‐dependent were conducted at 5% shear strain to ensure properties were measured within the LVR while avoiding signal noise at lower strain. The critical strain (*γ*_c_) data showed that as the concentration of Alg and CMCS increased, the LVR decreased.

To demonstrate the effect of CMCS on the loss tangent (tan(*δ*)) of the composites, additional solutions containing 2%, 3%, and 4% w/v Alg were compared to the composite solutions. As all the tested solutions were viscoelastic fluids, the tan(*δ*) values were greater than 1. The 2|0 solution had the highest tan(*δ*) of 3.58, while 4|4 had the lowest value of 1.69 (Figure 2c). As the concentration of both Alg and CMCS increased, the loss tangent decreased. An unexpected property of CMCS presented itself in these tests, where adding low concentrations of CMCS to 4% w/v Alg, tan(*δ*) of 1.90, increased the tan(*δ*) of 4|1 and 4|2 to 2.02 and 1.93, respectively, contrary to the decreasing trend observed in the lower Alg concentrations. This indicates that adding low concentrations of CMCS to a relatively high concentration of Alg decreases the elastic behavior of the viscoelastic solutions compared to a solution only containing Alg, but the overall trend of the elasticity increases as CMCS concentration increases.

### 3.2 Frequency‐Dependent Behavior of Alg‐CMCS

**FIGURE 3:**
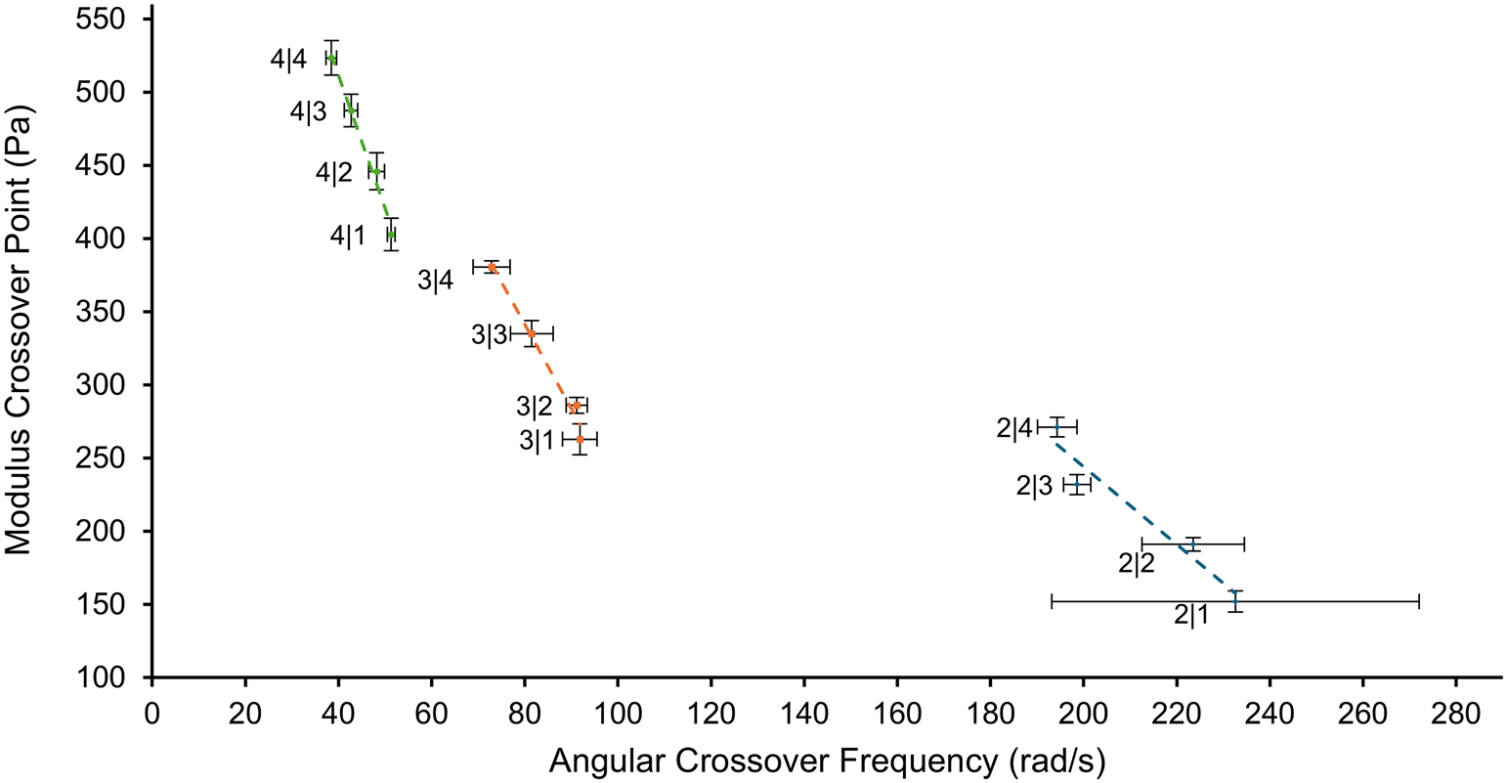
Crossover point means shown with trendlines, and horizontal and vertical error bars representing standard deviation.

For viscoelastic shear‐thinning fluids, the angular crossover frequency (ACF) is the point at which solutions temporarily harden from a viscous liquid state to a solid gel, while the crossover modulus (CM) is the magnitude of both moduli since this transition occurs when *G*′ = *G*″. Composite solutions containing 4% w/v Alg had the lowest crossover frequency, where 4|4 had an ACF of 38.5 rad s^−1^ or 6.1 Hz, and a CM of 128.2 Pa, which was 2.8 times higher than the *G*″ in the LVR, shown in Figure 3 below. Solutions containing 2% w/v Alg had the highest ACF, and 2|1 had an ACF of 232.6 rad s^−1^ or 37.0 Hz, with a CM of 152.0 Pa, which was 7.6 times higher than the *G*″ of the same solution in the LVR. As with previous properties, the ACF and CM of the composite solutions were more affected by Alg concentration than CMCS. As the concentration of both materials increased, the CM increased, but the ACF decreased. 2|4 and 3|1 had similar CM—271.2 and 262.9 Pa, respectively—but the difference in ACF was large—194.3 and 91.8 rad s^−1^, respectively. However, as the concentration of Alg increased, the difference in ACF between solutions decreased. For example, 3|4 and 4|1, with similar CM of 380.5 and 402.8 Pa, respectively, had ACF of 72.9 and 51.3 rad s^−1^, respectively—a marked decrease in the difference between the ACF.

### 3.3 Viscosity Models for Alg‐CMCS

The recorded data for apparent viscosity (*μ*), changing with shear rate ($\dot{\gamma }$), was fitted using MATLAB to extract the variables for each composite solution, creating a consistent Carreau‐Yasuda (C‐Y) model for each sample. Using Equation S1, the zero‐shear viscosity (*μ*_0_), infinite‐shear viscosity (*μ*_∞_), time constant (*λ*), power law exponent (*n*_CY_), and transition index (*a*) were obtained are summarized in Table 1 below.

**TABLE 1:** C‐Y model variable estimates for each composite solution studied.

Sample	Zero‐shear viscosity, *μ*_0_ (Pa s)	Infinite‐shear viscosity, *μ*_∞_ (Pa s)	Time constant, *λ* (s)	Power law exponent, *n*_CY_	Transition index, *a*
2|1	4.2	0	0.036	0.330	0.801
2|2	6.2	0	0.016	0.094	0.630
2|3	8.2	0	0.057	0.365	0.776
2|4	11.4	0	0.048	0.290	0.678
3|1	16.8	0	0.068	0.277	0.808
3|2	18.7	0	0.062	0.250	0.752
3|3	26.1	0	0.076	0.251	0.719
3|4	35.2	0	0.093	0.260	0.692
4|1	41.9	0	0.112	0.264	0.819
4|2	55.2	0	0.126	0.254	0.772
4|3	71.5	0	0.153	0.263	0.744
4|4	92.2	0	0.192	0.268	0.723

The *μ*_∞_ values obtained from MATLAB were all zero or close to zero, indicating that viscosity reaches zero resistance to flow at infinite shear. As the concentration of the composite increased, again with Alg influencing the properties more than CMCS, the viscosity at zero‐shear and the time constant increased, the power law exponent remained relatively constant, and the transition index decreased.

**FIGURE 4:**
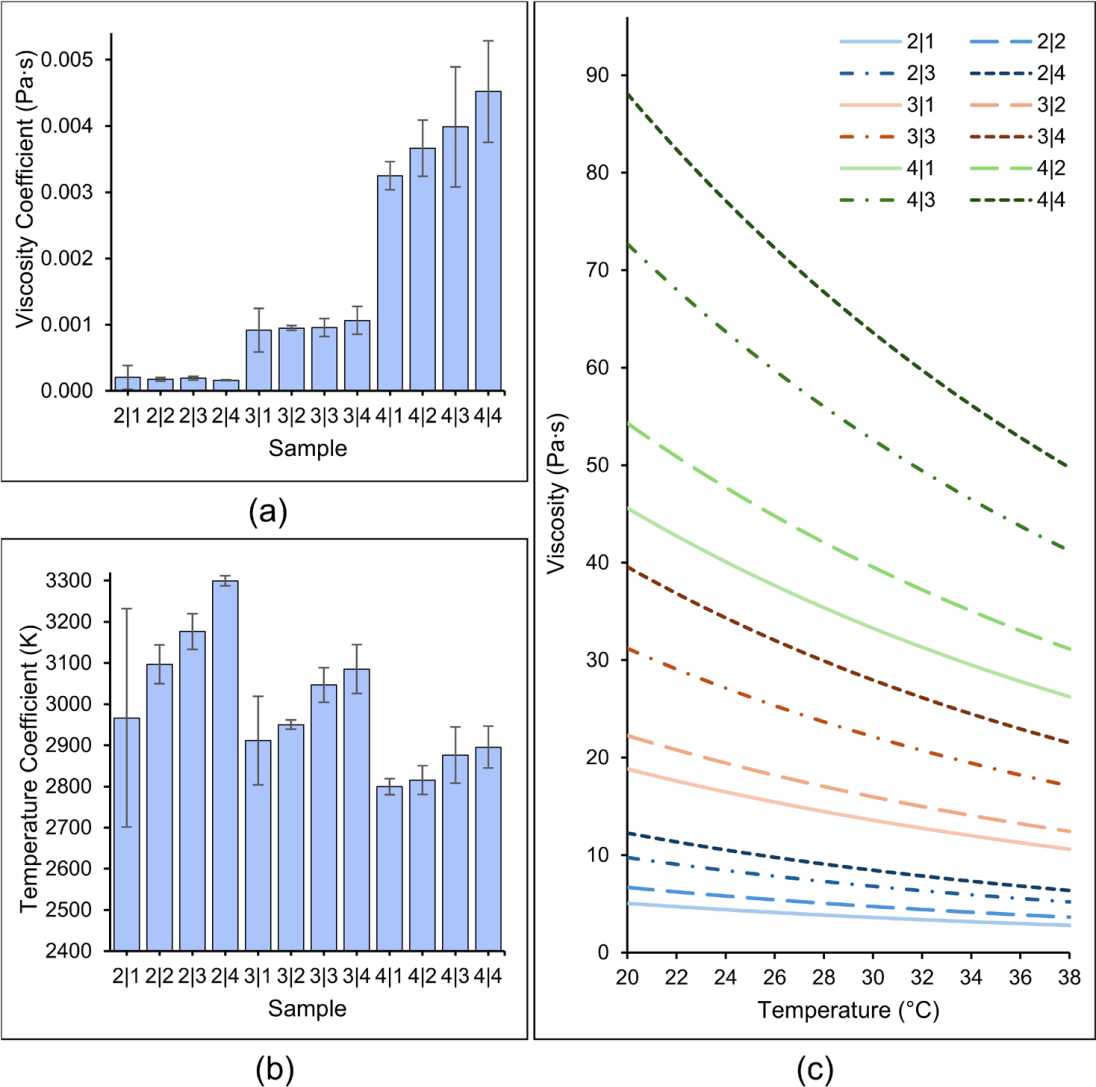
(a) Viscosity coefficient estimates with error bars representing the upper and lower bounds of the 95% confidence interval (CI), (b) temperature coefficient estimates with 95% CI bars, and (c) temperature‐dependent viscosity models created using the coefficients. Composite solutions are grouped by Alg concentration color, differentiated by shading and line type.

MATLAB was also used to fit the temperature‐dependent viscosity data to a curve created using Equation S2. Figure 4 below shows the modified Arrhenius equation coefficients and the model created with the recorded data for each solution at low constant shear rate. The viscosity coefficients for 2% and 3% w/v Alg‐containing solutions exhibited minimal variation, while 4% w/v Alg‐containing solutions displayed a clear increase in variation as the CMCS concentration increased. Similarly, for the temperature coefficient, as the concentration of CMCS increased, the temperature coefficient also increased. Increasing the concentration of Alg had the opposite effect: as the concentration increased, the temperature coefficient decreased. For instance, 2|1 had a viscosity coefficient (𝜇_𝑐_) of 2.03E−04 Pa s and a *β* of 2966.6 K, 3|1 had a 𝜇_𝑐_ of 9.16E−04 Pa s and a *β* of 2911.4 K, and 4|1 had a 𝜇_𝑐_ of 3.25E−03 Pa s with a *β* of 2799.4 K.

### 3.4 Printability Flow Rate Models and Calculated Printing Parameters

**FIGURE 5:**
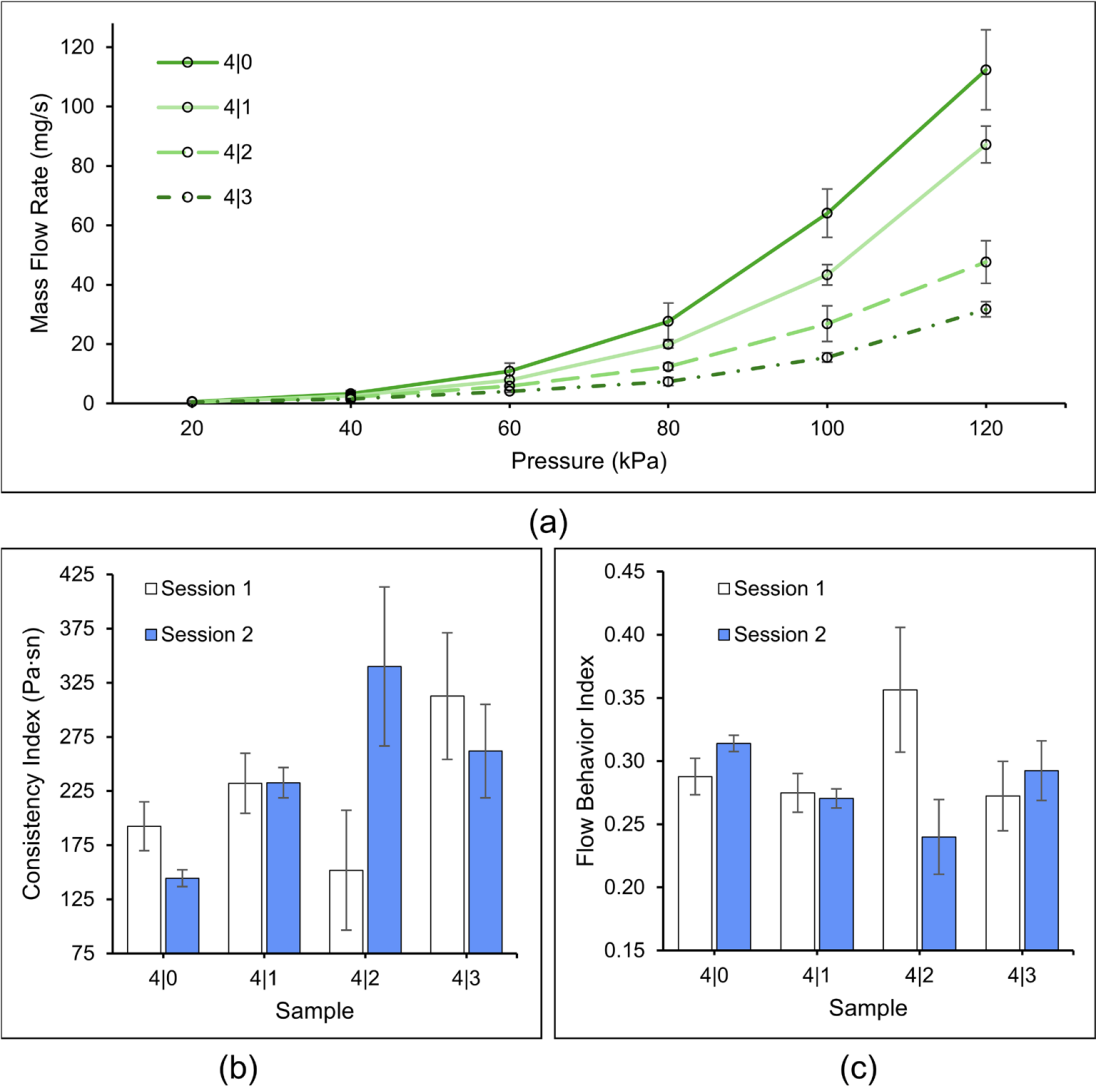
(a) Recorded mass flow rate data with standard deviation bars, (b) consistency index values with bars representing the bounds of the 95% CI of the estimate, and (c) flow behavior index values with 95% CI bars.

By modeling the flow behavior of the solutions, the need for trial‐and‐error printing to obtain scaffolds for printability analysis was eliminated, thereby saving time and conserving materials. From the recorded data, as the concentration of CMCS increased, the mass flow rate decreased in the four synthesized solutions, as seen in Figure 5a below. *K* and *n* values in Equation (1) were determined for each solution by using MATLAB curve fitting, as shown in Figure 4b,c. The consistency index, *K*, values reinforced the rheological property results, indicating that increasing the concentration of CMCS led to higher viscosity, reducing the mass flow rate. The flow behavior index, *n*, slightly decreased as CMCS concentration increased, indicating that the materials became more shear‐thinning under induced stress.

Using the values for *K* and *n*, Equations (1) and (2) were used to calculate the printing speed required to achieve the designed filament diameter of 200 μm for each solution from 20 to 120 kPa. To avoid excessive dithering of the printed filaments, printing speed was kept under 60 mm s^−1^. The calculated speed and the corresponding pressure used to print scaffolds are summarized in Table 2 for each solution. As the concentration of CMCS increased, the printing pressure also increased to compensate for the increased viscosity of the solutions.

**TABLE 2:** Calculated printing speeds for the two printing sessions required to create a filament with a 200 μm diameter.

Sample	Session 1		Session 2	
	Printing pressure (kPa)	Printing speed (mm s^−1^)	Printing pressure (kPa)	Printing speed (mm s^−1^)
4|0	30	25.5	30	47.2
4|1	40	43.3	40	45.8
4|2	40	55.0	50	39.8
4|3	50	34.2	50	48.2

*Note:* Speed was calculated separately for each session, and the pressure was adjusted accordingly.

### 3.5 Filament Fidelity Indexes

**FIGURE 6:**
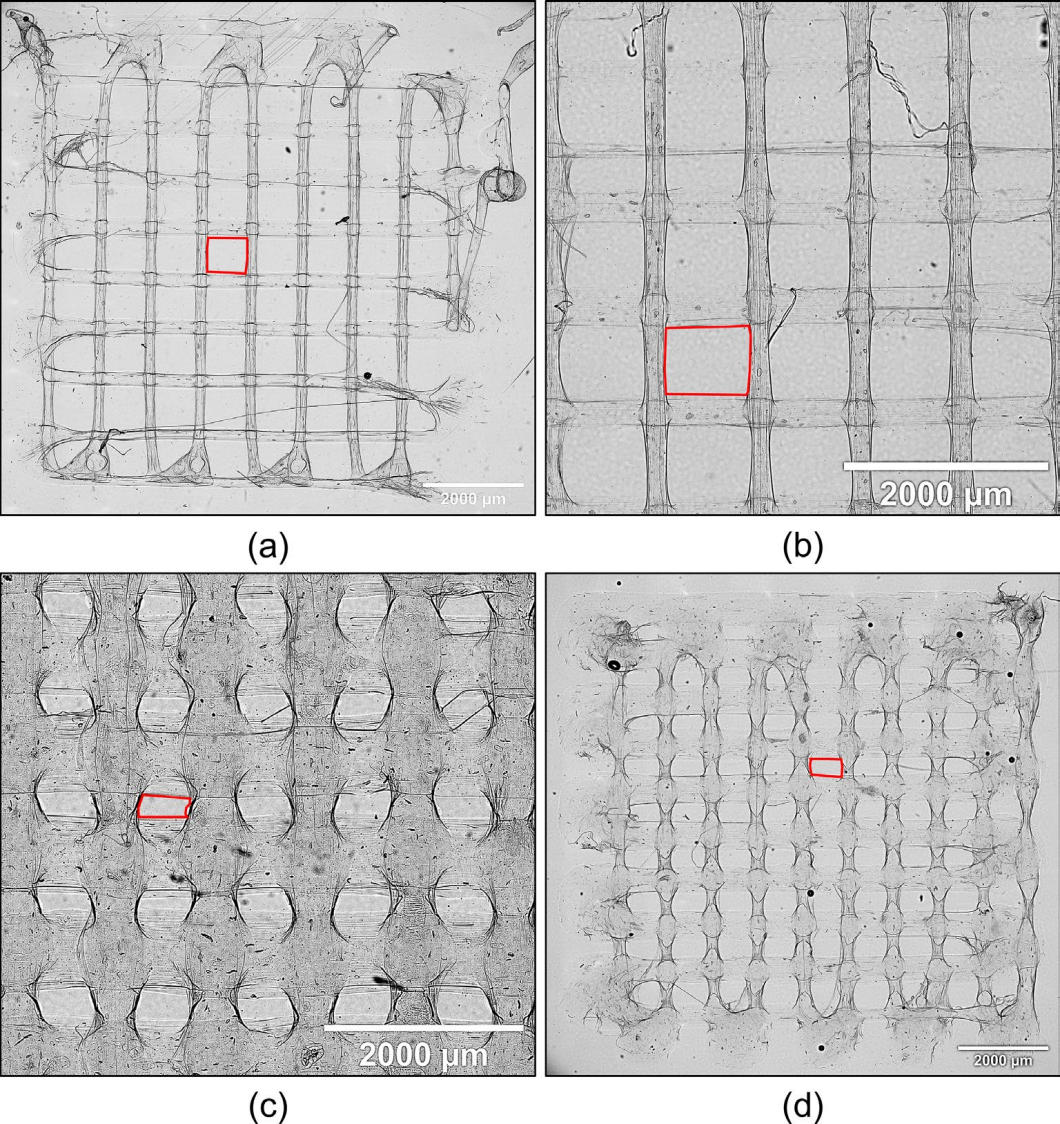
Top‐down, brightfield images of the scaffolds printed for printability characterization; traced in red are the general shapes of the pores. (a) A 4|0 scaffold with thin filaments but large discontinuities around the edges, (b) a closer view of a 4|1 scaffold, similar in appearance to 4|0 but with better layer printing, (c) the very large filaments and small pores of a 4|2 scaffold, and (d) a 4|3 scaffold, similar in appearance to 4|0 but with larger filament widths.

Using brightfield microscope images, the indexes of WDR, OLI, PrI, and PrF were calculated for the four solutions. Filament fidelity indexes closest to 1 indicated that the printed scaffold most accurately matched the designed scaffold compared to the other solutions. The indexes of each solution and in each category were calculated as the median of the values for each scaffold for statistical comparison. 4|0 had the closest filament fidelity indexes in WDR and OLI, with index values of 1.76 and 0.96, respectively, while 4|1 had the closest accuracy in the indexes of PrI and PrF, with values of 0.46 and 1.03, respectively. Examples of printed scaffolds are displayed in Figure 6 below.

**FIGURE 7:**
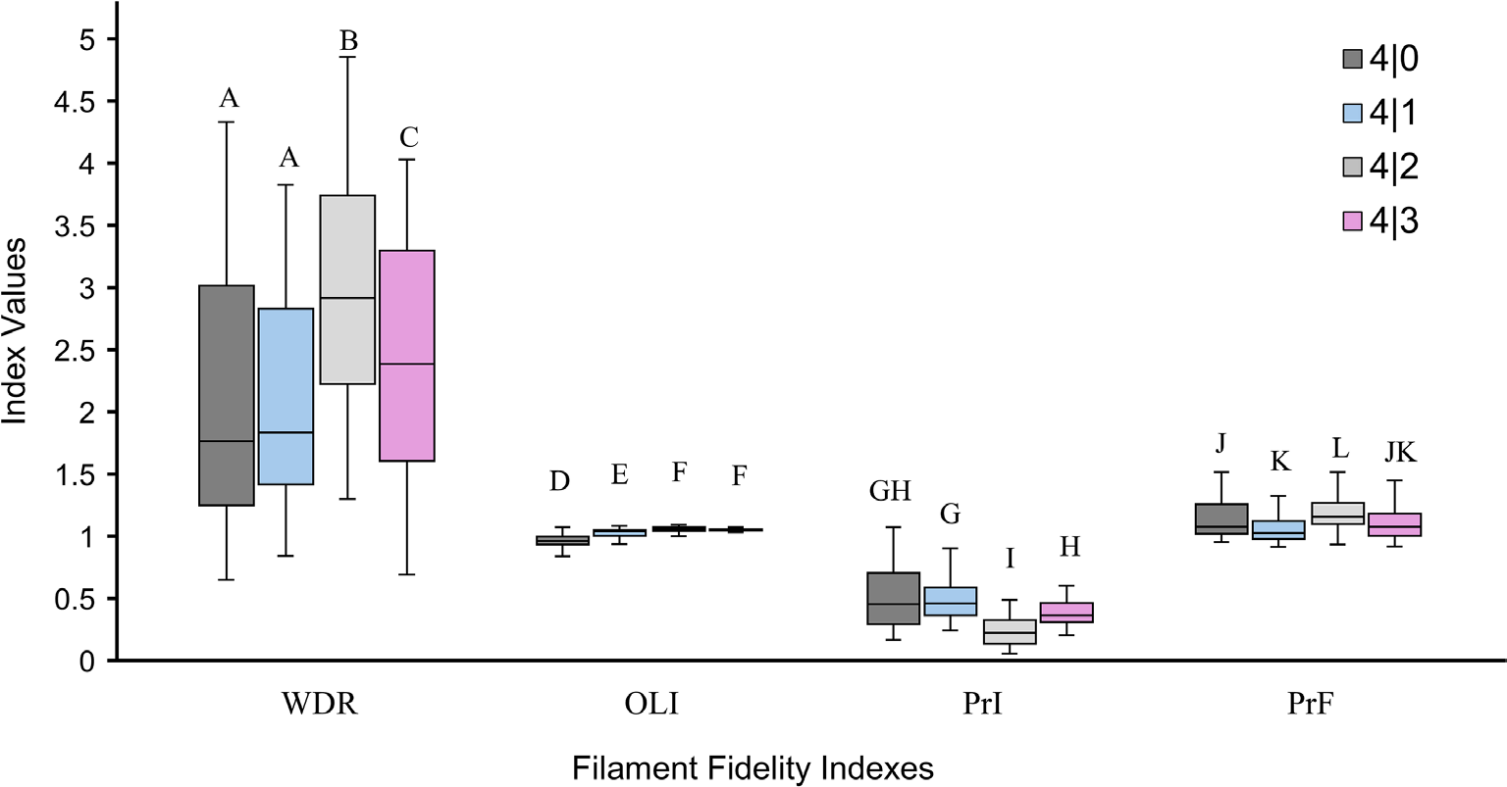
Box plots showing the median values and interquartile ranges of the filament fidelity indexes. Bars represent upper and lower values of each index excluding outliers. Letters represent significant differences between the medians of each solution (*p* < 0.05); shared letters represent non‐significant differences (*p* > 0.95).

For each of the four indexes, the Kruskal–Wallis test—with 3 degrees of freedom for each index (df = 3)—indicated that there were significant differences between the medians of the printed scaffolds for all the solutions: WDR (*χ* ^2^ = 135.49, *p* < 2.2E−16); OLI (*χ* ^2^ = 1330.01, *p* < 2.2E−16); PrI (*χ* ^2^ = 83.17, *p* < 2.2E−16); and PrF (*χ* ^2^ = 40.3, *p* < 9.2E−09). The results of the calculated indexes and corresponding statistics are summarized in Figure 7 below.

Dunn's test revealed that for the WDR, only the filaments of 4|0 and 4|1 scaffolds did not significantly differ in their median values of 1.76 and 1.83, respectively (*Z* = −0.747, *p* = 0.45). All the other scaffolds significantly differed (*p* < 0.05) in their widths from each other and from the designed diameter of 200 μm. The 4|3 scaffolds had a WDR index of 2.39, and the 4|3 scaffolds had the largest WDR index at 2.92. The trend of the WDR index slightly increased as the concentration of CMCS increased.

The outer size of the scaffolds, when compared to the design, was best overall for 4|0 scaffolds with an OLI of 0.96. 4|1 had an OLI of 1.04, but the difference between 4|0 and the ideal value of 1.0 was closer by 0.001. Statistically, only 4|2 and 4|3 scaffolds were not significantly different (*Z* = 0.708, *p* = 0.48) with OLI of 1.06 and 1.05, respectively. With index values close to 1.0, all the different biomaterial solutions demonstrated the ability to replicate the outer shape and dimensions of the design to a high degree of accuracy. 4|0 scaffolds had an OLI under 1, while solutions containing CMCS had an OLI index over 1.

When looking at PrI, the medians of 4|0 and 4|1 (*Z* = −0.757, *p* = 0.45) and 4|0 and 4|3 (*Z* = 2.175, *p* = 0.06) were not significantly different. PrI is tied to the size of the filaments, which is why the trend of the values mirrors the trend of the WDR, but instead of increasing as the concentration of CMCS increases, PrI decreased.

The pores of the other scaffolds became more rectangular as the PrF deviated from 1. With a PrF index of 1.03, 4|1 scaffolds had the greatest ability to retain a square‐shaped pore over the scaffolds printed with the other biomaterial solutions. The medians of 4|0 and 4|3 were not significantly different (*Z* = 1.485, *p* = 0.14), 4|1 and 4|3 were not significantly different (*Z* = −2.177, *p* = 0.06), but despite 4|0 and 4|3 having the same PrF of 1.08, 4|0 and 4|1 medians were statistically significant (*Z* = 3.662, *p* = 0.001). From the results, there was no apparent trend for the fidelity of the pore shape as CMCS concentration increased.

## 4 Discussion

### 4.1 Critical Shear Strain and the LVR

Characterizing the LVR is important for strain‐dependent tests since this is the region in which stress and strain are directly proportional. This means that measured material properties like *G*′, *G*″, and tan(*δ*) will have consistent values. The Alg‐CMCS composite solutions had high LVRs, which means that the material had a longer time in‐region where strain and stress can be calculated quantitatively rather than relying on empirical models. With a high LVR, this also means that any experiments that control strain, like frequency‐dependent properties of materials, can be conducted safely within the LVR, decreasing the instability of obtained results. This is why any subsequent strain‐dependent, dynamic shear tests were conducted at 5% strain so that any unpredictable fluctuations in the strain would not affect the recorded data and there was no risk of exceeding the $\gamma_{c}$ during experimentation. As the concentration of both Alg and CMCS increased, the upper limit of the LVR decreased and Alg displayed a greater influence than CMCS, for example, 2|4 having a higher LVR than 3|1 despite having more dry material mass.

### 4.2 *G*′ and *G*″

*G*′ and *G*″ make up the complex modulus (G*) and reflect the dual viscous and elastic nature of the Alg‐CMCS composite solutions. From Figure 2a, for the composite solutions, Alg had a more influential effect on the increase of both *G*′ and *G*″ than CMCS. For example, the *G*′ and *G*″ of the three solutions containing 4% w/v CMCS—2|4, 3|4, and 4|4—did not exceed the next level of Alg concentration—3|1 and 4|1. 2|4 had a *G*′ of 15.5 ± 0.7 Pa and a *G*″ of 41.7 ± 1.6 Pa; since this solution has a total dry material mass concentration of 6% w/v, it would be logical to think that 2|4 would have higher moduli than 3|1, which only has a total material concentration of 4% w/v. However, 3|1 had higher *G*′ and *G*″ values, 25.6 ± 2.0 Pa and 64.1 ± 4.2 Pa, respectively, indicating that Alg had a higher influence on these properties than CMCS.

The increase in *G*′ demonstrated that the solution became increasingly solid, but *G*″ also increased, which counteracted the solidity by adding some liquid behavior. The increasing storage modulus led to an increase in the viscosity of the solutions as both Alg and CMCS concentrations increased. For 3D printed solutions, it is better to have a higher *G*′ so that material retains its shape post‐extrusion when resting on the printing stage. Generally, this means that higher viscosity solutions have better printing resolution [34]. However, the balance between *G*′ and *G*″ is also critical for extrusion printing because the material must flow under shear forces but also retain its shape once deposited. For all composites, both *G*′ and *G*″ increased as the concentration of both Alg and CMCS increased, but the slope of the trend between Alg concentration groups—2% = 2.2, 3% = 1.6, and 4% = 1.3—decreased as the concentration of Alg increased. This indicates that increasing polymer concentrations will eventually lead to extremely high viscosity solutions with limited flow, requiring much higher pressures to print. This trade‐off highlights the importance of adjusting polymer mixture ratios to balance print fidelity with extrusion performance, especially in biomedical applications like tissue scaffolds where structural integrity and resolution are both essential.

### 4.3 Loss Tangent Characterization

The loss tangent, tan(*δ*), is another measure of the viscoelastic characteristics of a solution, and within the LVR, tan(*δ*) remains constant. However, as the system increases in strain, tan(*δ*) also increases. This means that the tan(*δ*) of the Alg‐CMCS solutions exceeded 1 at higher strains, showing that composites behaved less like elastic solids and more like viscous liquids. This is because the inner microstructures and bonds of the samples are deformed as the strain continues to increase, allowing the solution to flow more easily. In this context, more viscous means the samples act more like a liquid than a solid, rather than simply implying that the solution flows less readily.

Since tan(*δ*) is mathematically the ratio of *G*″ to *G*′, the moduli help to illustrate this point (Figure 2a) since, for all solutions, *G*″ dominated *G*′, reflecting the phase of the solutions as a liquid in the real world. However, as the concentration of both Alg and CMCS increased, this ratio decreased from around 3.5 down to 2 times the *G*′, representing a change to more elastic behavior. If the concentration of Alg and CMCS were increased beyond 4% w/v, there would be a further increase to a point where tan(*δ*) would be close to 1, the transition point where the liquid hardens into a solid. The material would become more elastic, storing more energy, but the viscous behavior would still be dominant unless the material were crosslinked.

Interestingly, adding CMCS to 4% w/v Alg increased the tan(*δ*) for solutions with CMCS concentrations less than 3% w/v. A higher tan(*δ*) corresponds with a more easily flowing material, which was unexpected since more dry material was added. This behavior was not observed in 2% and 3% w/v Alg‐CMCS solutions. According to the results, low concentrations of CMCS create more slip in the composites when combined with higher concentrations of Alg, enhancing the ability to flow, while other rheological properties—*γ*_*c*_, *G*′, and *G*″—remained unchanged. This suggests that subtle composition changes can significantly affect flow behavior without altering the overall profile of the fluid. This is particularly relevant when selecting and tuning biomaterial formulations for extrusion 3D printing.

### 4.4 Frequency‐Dependent Behavior

The characterization of the frequency‐dependent behavior of biomaterial solutions is relevant for 3D extrusion printing of tissue scaffolds. This is because the material is subject to cyclic force every time a layer is printed and then goes to rest, an operation that is dependent on frequency. When exposed to an external force, the viscoelastic solutions behave more like a viscous liquid under slow cycles of force and more like an elastic solid when subjected to fast cycles of force. This behavior is linked to the behavior of tan(*δ*) and was observed using an oscillation frequency testing, detailed in Supporting Information.

The ACF is the frequency at which viscoelastic materials transition from a flowing viscous state to a solid gel‐like state, where tan(*δ*) = 1, after which point at higher frequencies, for shear‐thinning viscoelastic fluids, *G*′ > *G*″ and tan(*δ*) < 1. Measuring the ACF is important for extrusion printing to avoid unwanted hardening of the material, which would prevent extrusion purely because of the speed of each cycle at which each layer is printed.

The relationship between the ACF and the minimum print time each layer needs to exceed to prevent hardening is found by simply taking the inverse of the ACF, in units of Hz. However, it should be noted that even for the high‐viscosity solutions tested here with relatively low ACF, no current existing extrusion printers can achieve the speeds at which the theoretical minimum is calculated. For example, 4|4 with the lowest ACF of 6.1 Hz would have a minimum print duration per layer time of 163 milliseconds. With current filament resolution and overall sizes of the 3D constructs being printed, this minimum time is easily surpassed. That said, this parameter needs to be kept in mind for the future as the technology evolves and constructs become smaller, more intricate, and potentially print faster. Therefore, the ACF and CM can quantify the limits by which printing parameters, such as printing speed and printing pressure, respectively, should be bound. In terms of an ideal printable solution, the solution should have a high ACF, which means a lower minimum printing duration or inversely a higher maximum allowable printing speed. A solution with a higher CM would possess the ability to withstand a higher threshold of force before gelling occurs, which would prevent clogging of the needle and could also protect cells from damaging forces if directly incorporated in the material.

### 4.5 Apparent Viscosity Models

When the recorded data were preliminary curve‐fitted with different models—Cross, Carreau, Carreau‐Yasuda, Sisko, and Williamson models—in both the TRIOS software and MATLAB, the C‐Y model best described the observed three‐region viscosity profile. According to the results summarized in Table 1, all composite solutions showed that at infinite shear, viscosity achieved effectively zero flow resistance. However, in extrusion printing applications, the rate and magnitude of shear would likely never reach this high, no matter how far extrusion bioprinting evolves. At high printing pressure, viscosity would decrease to extremely low levels due to the shear‐thinning property of the solutions but would never truly reach zero.

The composite solutions exhibit three regions of viscosity, which are well captured by the C‐Y model: an initial Newtonian region at rest and very low shear rate that transitions to a power law region between low and high shear rates, and finally a second Newtonian region at high to infinite shear. This behavior is particularly relevant for extrusion printing because the material should be stable at rest, flow with ease under induced shear, and recover quickly post‐extrusion to have good printability.

Delving further into the transition index of the composite solutions, adding CMCS to Alg decreased the width of the initial Newtonian zone, while increasing Alg concentration slightly increased the width. This behavior contrasts with all previous properties where Alg and CMCS would act in concert, either increasing or decreasing the relevant property together. The decrease in width shows that CMCS reduces the magnitude of shear rate that the solutions can withstand before flow begins, potentially making the material more responsive to extrusion forces while maintaining stability at rest. Simultaneously, the time constant, or characteristic time, which increases as the concentration of Alg and CMCS increases, indicates that higher viscosity solutions take longer to transition from a Newtonian to a power law fluid [35]. As the composite concentration increased, the power law exponent remained relatively unchanged, meaning that increasing shear rate affected the viscosity of the solutions similarly and at the same rate of shear.

Like the previous rheological properties, except the transition index, increasing Alg concentration had a more pronounced effect on the viscosity of the solutions than adding more CMCS. Adding CMCS increases the viscosity of each subsequent solution in a fairly linear manner, whereas increasing Alg concentration increased the viscosity in an inverse exponential way. Because of the diminishing impact of Alg concentration on viscosity, there may be higher, untested concentrations of Alg where viscosity would be evenly affected by both Alg and CMCS. However, this concentration may not be favorable for cell functions, and experiments need to be conducted to study the effects of this heightened biomaterial concentration on cell viability as the effects vary between cell types [36, 37, 38].

### 4.6 Temperature‐Dependent Viscosity

The sample replicates were reduced to *n* = 3 due to limited material availability, and all data for each sample were fitted in MATLAB with Equation S2, a modified Arrhenius equation, to find the viscosity and temperature coefficients. For the viscosity coefficient, CMCS increased the value only slightly at lower concentrations of Alg but increased significantly at 4% w/v Alg. Alginate affected the viscosity coefficient in more pronounced blocks than CMCS, which was expected based on the previous apparent viscosity results. Additionally, as the overall concentration of the composites increased, the variation in the lower and upper bounds of the estimates increased, indicating greater uncertainty at higher viscosities.

The temperature coefficient presented similar behavior to the transition index of the apparent viscosity, with material concentration working in opposition to affect *β*: increasing CMCS concentration increased the temperature coefficient, while increasing Alg concentration decreased the temperature coefficient. Within Alg groups, CMCS also introduce a clear gap between 2% and 3% w/v groups, for example, 3|1 and 3|2 had a difference of 39 K, 3|3 and 3|4 had a difference of 39 K as well, but the jump from 3|2 and 3|3 was 96 K. This behavior was present in all three Alg concentrations, although at 2% w/v Alg, the gap was smaller than the difference between 2|1 to 2|2 and 2|3 to 2|4—opposite of the increase in the gap for 3% and 4% w/v Alg. This gap indicates that there is a critical CMCS concentration between 2% and 3% w/v that affects the temperature coefficient of the composite solutions more than the increase between 3% and 4% w/v CMCS.

Looking at Equation S2, *β* represents the ratio of the activation energy (*E*_a_) to the universal gas constant (*R*) and reflects as the energy barrier that must be overcome for molecular flow to progress. A decrease in *β* means that less thermal energy is needed to transition the solutions to flow more readily, lowering the *E*_a_. This trend was observed with increasing Alg concentration, suggesting that alginate reduces the thermal resistance of the composites, similar to other polymer systems in the literature where increased polymer concentration also led to reduced activation energy [39]. Conversely, CMCS increased *β*, indicating a rise in activation energy and greater thermal resistance [40]. This property could be advantageous in bioprinting applications, where both printing and storage temperatures can influence the rate of material degradation [41]. Therefore, CMCS could help stabilize against excessive deformation and reduce the degradation rate of Alg‐based scaffolds. As with *G*′ and *G*″, the balance between the advantages and disadvantages of high and low *E*_a_ must be considered during material selection. Materials with lower activation energy may flow more easily at higher temperatures, reducing the extrusion pressure and potentially improving cell viability. On the other hand, materials with higher *E*_a_ may offer better thermal stability, which would support the long‐term structural integrity of the printed scaffold.

The Arrhenius coefficients can be used to model the viscosity behavior of each solution with respect to temperature, as seen in Figure 4c. This is helpful in visualizing the models that could predict how each composite solution will behave under different environmental conditions. As discussed in relation to activation energy, for extrusion printing, adjusting the temperature based on the material properties can reduce the pressure required to dispense material, which in turn can improve cell viability and printability [42, 43].

### 4.7 Flow Rate Models and Printing Parameters

The visual representation of the fitted *K* and *n* values in Figure 5b,c provides insight into the rheological properties of the materials, consistent with the previous experimental results. As the concentration of CMCS increased, the *K* values rose, while the *n* values remained relatively stable. The large variance in data for 4|2 and 4|3 is attributed to the large temperature fluctuation from 23°C to 31°C in the surrounding environment during the printing sessions. Since *K* represents the consistency index, with the same units as viscosity, the value can be used to describe the viscosity of the solution. It can be seen above that as the concentration of CMCS increased, the viscosity of the solution increased, reinforcing the results of rheological characterization. In terms of *n*, Figure 5c shows that the flow behavior index for all solutions did not greatly change with the addition of CMCS, the large variance in 4|2 being the exception due to the difference in printing conditions. This means that the shear‐thinning behavior was not significantly affected by the addition of CMCS.

By using the printing parameters from the results of flow modeling, two‐layer scaffolds were printed for later printability analysis. These two parameters are particularly important if cells are incorporated in the biomaterial solution. While the pressure range used here was relatively low, the effect of extrusion pressure, combined with needle diameter, on cell damage has been presented by multiple authors [42, 44, 45, 46]. The consensus is that as extrusion pressure increases, cell viability decreases. The maximum pressure that cells can withstand before damage occurs is cell dependent. Similarly, printing speed can create compressive and tensile forces on the filament, which need to be tested with specific cells to observe the ability of the cells to resist permanent damage.

### 4.8 Scaffold Repeatability

Repeatability, in the context of scaffold printing, refers to the ability to produce scaffolds consistently and successfully across multiple prints. To ensure filament attachment to the bottom of the well, the *z‐*offset for the printer needed to be set to −0.14 ± 0.02 mm. For reference, a *z*‐offset of −0.20 mm touched the surface of the wells. If the height was set higher, filaments remained stuck on the needle instead of the surface, negatively affecting the repeatability. This issue arises because the density of the scaffolds and the crosslinking solution are similar, creating buoyancy in the scaffold materials. While this property helps reduce the gravitational spread of the printed filaments, the similarity in density can make scaffold formation more difficult [12, 47]. This was more apparent for the 4|0 solution that did not contain CMCS, which had a lower density than the composite solutions (1.02 g mL^−1^ vs. 1.1 g mL^−1^), resulting in reduced repeatability. Of the 24 wells used to print 4|0 scaffolds, only 5 (~21%) contained successfully produced two‐layer scaffolds that were viable for further printing.

The discrepancy between scaffold repeatability and the number of scaffolds used for printability analysis exists because the indexes focused on quantitative measurement of filaments, pores, and outer length, rather than the qualitative assessments of scaffold quality and integrity. For the composite solutions, scaffold repeatability significantly improved for 4|1, 4|2, and 4|3, which achieved successful scaffold formation in 75%, 62%, and 62%, respectively, of the wells. This demonstrates that adding CMCS to Alg increased scaffold repeatability by approximately three times.

This improvement in repeatability has important implications for research and clinical applications. In tissue engineering, consistent scaffold fabrication is essential for reproducibility in experimental studies and for scaling up production for clinical use, where timely access to functional scaffolds could be lifesaving. High repeatability also reduces material waste, improves throughput efficiency, and ensures that printed scaffolds meet the research or patients' needs, which are critical factors for regulatory approval and quality control. Clinically, reliable scaffold platforms support uniform cell seeding or incorporation, predictable mechanical properties, and consistent degradation profiles, all of which contribute to better patient outcomes. As tissue engineering advances toward personalized and large‐scale applications, materials that support high repeatability will be one of the key factors bridging the gap between laboratory research and clinical translation.

### 4.9 Filament Fidelity Indexes

Interestingly, filaments printed along the *y*‐axis were closer to the designed diameter of 200 μm than filaments printed in the *x* direction. This can be explained by the need for a much lower first layer; the *x*‐axis filaments were created by the decreased *z*‐offset. As the filaments were extruded, they were slightly flattened to adhere to the printing surface. Because subsequent layers followed the programmed layer height, they were not as flattened as the first layer. The widths of the filaments, however, were still comparatively large when looking at the designed diameter. The increasing viscosity as CMCS concentration increases may also exacerbate this because lower speeds are required to print filaments of the same diameter at the same pressure as viscosity increases. This, in addition to the larger needle outlet diameter, is the reason the WDR is more than double when compared to the designed diameter. This may be rectified by adding more pre‐flow—some material is extruded before the scaffold layer begins printing—and increasing the *z*‐offset.

Despite using the bioplotting technique—directly printing in a crosslinking media—the material was still able to spread because of the relatively low concentration of the crosslinking solution and gravitational effects on the material. Additionally, due to the mechanics of crosslinking, swelling of the material occurred because of a difference in osmotic pressure inside and outside of the rapidly crosslinking filament. This caused the filament to retain some crosslinking solution, resulting in slightly more swelling of the filament widths [48, 49]. Since the composite materials had higher WDR and OLI indexes than the solution containing only Alg, this could demonstrate that CMCS increases the swelling experienced by the material. This is likely because CMCS does not contribute to any crosslinking groups in the presence of calcium ions and would slightly inhibit the speed of the crosslinking process.

For the PrI, while the smaller pore sizes did not match the design, they contributed to better structural integrity since the intersection points between the layers had more surface area to connect. Reflected by the PrF results, qualitatively, the 4|1 scaffolds had the most square‐shaped pores, while the pores for 4|2 and 4|3 scaffolds were very small and slit‐like, as indicated by their low PrI indexes of 0.22 and 0.36, respectively. The 4|0 scaffolds had larger but still rectangular pores, as shown by the combined PrI and PrF results. Maintaining the shape of the pores is important as it indicates that the material can accurately replicate the design. For 4|1, while the sizes of the pores were almost half the designed size, the material could create almost perfect squares like the design. As indicated by the low PrF of 4|1 scaffolds, it is highly likely that the biomaterial could replicate most designed internal structures and different filament orientation angles the best out of all the materials printed here.

It should be noted that while 4|0 scaffolds had the closest index value for OLI at 0.96, the filaments that were measured were often incomplete or not attached to the rest of the scaffold. This irregularity is not reflected in the OLI results because the printed scaffold was only formed by two layers, and the inconsistent printing was not enough to disrupt the formation of these layers. Additionally, the repeatability of the scaffolds indicates that this biomaterial does not have a good success rate when used to print more than two layers. For this reason, and reinforced with the analysis above, combined with the fact that 4|0 and 4|1 were not statistically significantly different in the WDR index, 4|1 can be considered the best printable solution in all four measured indexes. Ultimately, this means that 4|1 was the optimal solution to print scaffolds most accurately when compared with the design.

## 5 Conclusions

Alg‐CMCS composite solutions are used to 3D print tissue scaffolds that help repair damaged tissue. However, the impact of Alg and CMCS on the rheological properties and printability of these composite solutions has not been discovered and documented, necessitating a laborious trial‐and‐error printing process. This study investigated the effects of Alg and CMCS on the rheological properties and printability of the composite solutions to rigorously design the printing process. Using dynamic and steady shear tests to analyze the rheology of the composites, it was observed that Alg had a greater influence on all rheological properties. But CMCS also contributed two notable properties to the composites: (1) at 4% w/v Alg, the elasticity of the composites containing low CMCS concentrations decreased below that of 4% w/v Alg‐only solutions, contrary to the general trend where increasing CMCS concentration increased the elasticity of the composites; and (2) CMCS was shown to increase the thermal resistance of the composite solutions, while Alg decreased the resistance. By implementing a novel flow rate‐based modeling approach to design the printing process, the printability of the composite solutions was examined using two‐layer scaffolds and quantified with four filament fidelity indexes. Based on OLI and WDR indexes, adding CMCS to Alg caused the outer shape and filament width of the composite scaffolds to be larger than the scaffolds that were printed with a solely Alg solution. However, it was observed that while 4|0 had the best OLI and WDR, 4|1 had better edge consistency, and the WDR did not significantly statistically differ between 4|0 and 4|1. Furthermore, 4|1 had the best PrI and PrF indexes; therefore, 4|1 could be considered the best printable solution in all four filament fidelity indexes. Finally, the addition of CMCS to Alg greatly increased the repeatability of scaffold printing by around three times when compared to scaffolds printed with only Alg. These results provide a deeper understanding of the biomaterial properties, emphasize the importance of material selection in scaffold printing, and support the use of modeling approaches to design the printing process, paving the way for more efficient and informed development of tissue scaffolds in future research.

## Author Contributions

**Xavier L. Tabil:** conceptualization, formal analysis, investigation, methodology, validation, visualization, writing – original draft, writing – review and editing. **Tate N. Cao:** funding acquisition, methodology, supervision, writing – review and editing. **Xiongbiao Chen:** conceptualization, funding acquisition, methodology, resources, supervision, writing – review and editing.

## Conflicts of Interest

The authors declare no conflicts of interest.

## Supporting information


**Figure S1:** Scaffold exclusively designed to examine printability.

## Data Availability

The data that support the findings of this study are available from the corresponding author upon reasonable request.
