# Supplementary material for: On the Rheological Properties and Printability of Sodium Alginate–Carboxymethyl Chitosan Composite Solutions for Tissue Scaffold Printing
Source: Biopolymers. 2025 Sep 24;116(6):e70050. doi: 10.1002/bip.70050 (PMC12457989; doi:10.1002/bip.70050)
Supplement: Supplementary file 1 — Figure S1: Scaffold exclusively designed to examine printability. [file BIP-116-e70050-s001.docx]

**Supplementary Material for:**

On the Rheological Properties and Printability of Sodium Alginate - Carboxymethyl Chitosan Composite Solutions for Tissue Scaffold Printing

Xavier L. Tabil^a*^, Tate N. Cao^b^, Xiongbiao Chen^a,c^

^a^ Division of Biomedical Engineering, College of Engineering, University of Saskatchewan, 57 Campus Drive, Saskatoon, SK, S7N 5A9, Canada

^b^ Ron & Jane Graham School of Professional Development, College of Engineering, University of Saskatchewan, 57 Campus Drive, Saskatoon, SK, S7N 5A9, Canada

^c^ Department of Mechanical Engineering, College of Engineering, University of Saskatchewan, 57 Campus Drive, Saskatoon, SK, S7N 5A9, Canada

*Corresponding authors:

Xavier L. Tabil, xavier.tabil@usask.ca; ORCID: https://orcid.org/0009-0003-8360-1839

Additional author information:

Tate N. Cao, tate.cao@usask.ca; ORCID: https://orcid.org/0000-0002-9159-5577

Xiongbiao Chen, xbc719@usask.ca; ORCID: https://orcid.org/0000-0002-4716-549X

**Supplementary Methods**

### Dynamic Shear Tests

All dynamic shear tests used the parallel plate configuration. To observe the viscoelastic behavior of the solutions, an oscillation amplitude test of 1–100% strain was applied logarithmically to the samples at a constant sinusoidal frequency of 1 Hz. To verify the linear viscoelastic region (LVR), the critical strain ($\gamma_{c}$) was found for each sample. This was determined by calculating a 5% drop in the storage modulus (*Gʹ*), which is more sensitive to changes in strain, and interpolating the corresponding strain value. The storage modulus, loss modulus (*Gʺ*), and tangent of the phase angle (tan(*δ*)), also known as the loss tangent, were characterized by averaging data from 1–10% strain. The frequency-dependent behavior of the materials, where the naturally liquid solutions harden at a certain frequency of shear, was also characterized by dynamic shear testing. To observe the frequency‑dependent behavior, a decreasing oscillation frequency test from 80–0.1 Hz was applied at a constant 5% strain. From this data, the intersection of *Gʹ* and *Gʺ* (*Gʹ* = *Gʺ*) was found, which corresponds to the crossover modulus (CM) and occurs at the angular crossover frequency (ACF).

### Steady Shear Tests

Steady shear testing was used to observe the viscosity response to both shear rate and temperature changes separately. The first test, a shear rate flow sweep, was used to characterize the apparent viscosity profile of the materials using the cone-and-plate configuration. Viscosity response was observed over an increasing shear rate from 0.01–250 s^-1^. MATLAB R2024a v24.1.0.2537033 was used to fit all the replicate data together using the Carreau-Yasuda (C-Y) fluid model, which was preliminarily found to fit most of the data best, with the following form:

$\mu=\mu_{\infty}+{\left( \mu_{0}-\mu_{\infty} \right)[1+{(\lambda\dot{\gamma})}^{a}]}^{\frac{\left( n_{CY}-1 \right)}{a}}$ (S1)

In the equation, $\mu$ is the apparent viscosity (Pa∙s), $\mu_{\infty}$ is the viscosity at infinite-shear (Pa∙s), $\mu_{0}$ is the viscosity at rest or zero shear (Pa∙s), $\lambda$ is the characteristic time constant of the shear-thinning transition (s), $\dot{\gamma}$ is the shear rate (s^-1^), $a$ is the transition index which accounts for region of transition from zero-shear to power law regions, and $n_{CY}$ is the power law exponent accounting for the slope in the power law region.

The second test, a temperature ramp, characterized the temperature dependence of the viscosity at a constant shear rate using the parallel plate configuration. Temperature was decreased from 38–20°C at a rate of 3°C min^-1^ at a constant shear rate of 1 s^-1^. MATLAB was used to fit the acquired data with a modified Arrhenius equation:

$\mu=\mu_{c}\cdot{exp}^{\frac{\beta}{T}}$ (S2)

where $\mu_{c}$ is the viscosity coefficient (Pa∙s), $\beta$ is the temperature coefficient (K) which is the ratio of activation energy (*E_a_*) to the universal gas constant (*R*), and $T$ is the temperature of the sample (K).

### Scaffold Design

Figure S1 below shows a 3D reconstruction of the scaffold design used to analyze printability for each biomaterial solution.


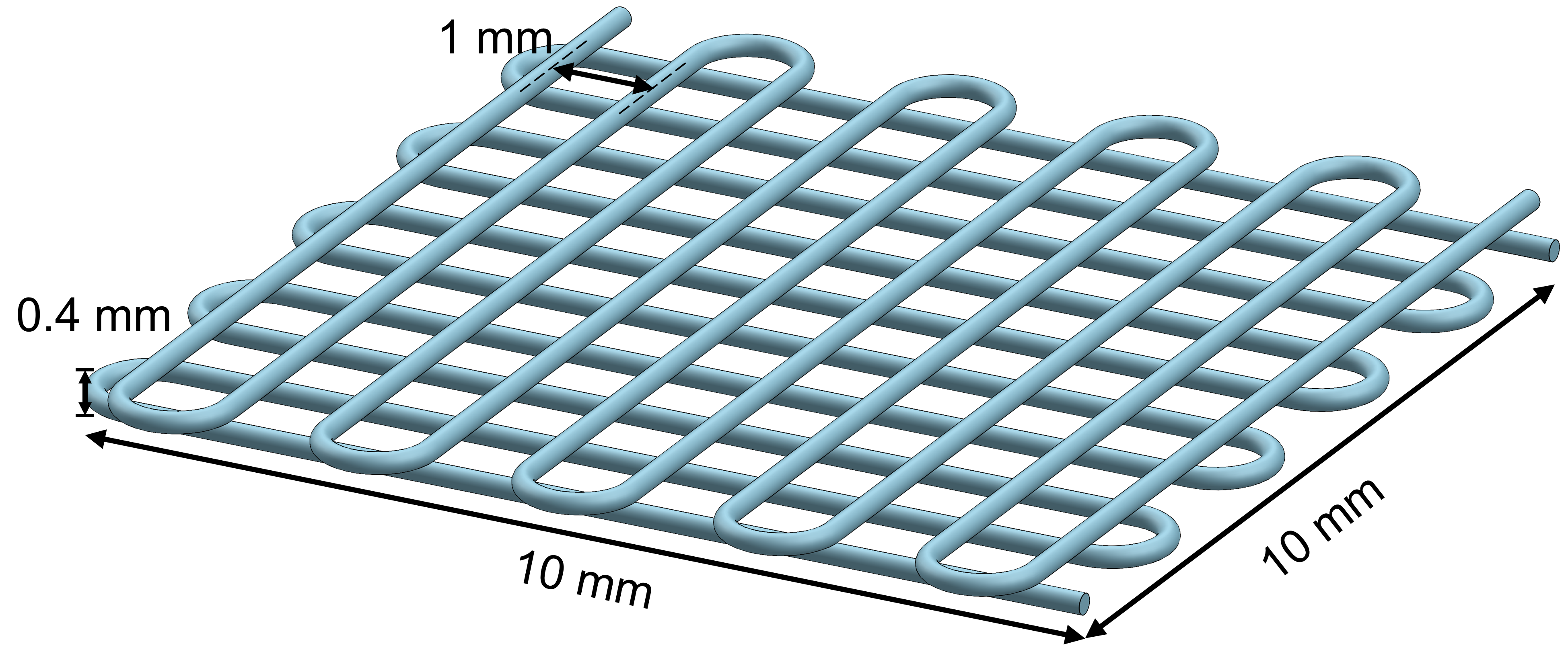


**Figure S1.** Scaffold exclusively designed to examine printability.
